# An Audit of the Completion of Physical Health Investigations for Patients Admitted to the General Adult Inpatient Wards in Mersey Care NHS Foundation Trust

**DOI:** 10.1192/bjo.2025.10605

**Published:** 2025-06-20

**Authors:** Vivian Mgbachi, Roweida Sammour, Declan Hyland

**Affiliations:** Mersey Care NHS Foundation Trust, Liverpool, United Kingdom

## Abstract

**Aims:** Individuals with severe and enduring mental illness are at increased risk of poor physical health and demonstrate reduced life expectancy of 15 to 20 years, compared with the general population. Despite being at increased risk of physical health comorbidities, such individuals are less likely to engage with physical health assessment and with required monitoring of any physical health comorbidity in the primary care setting. Admission to a psychiatric ward provides an opportunity to “screen and intervene”. Mersey Care NHS Foundation Trust has a policy for physical health assessment and monitoring following admission to the ward. This audit aimed to assess level of compliance with the policy on the Trust’s general adult inpatient wards.

**Methods:** The patient list for nine general adult inpatient wards in the Trust was obtained, forming a sample of 160 inpatients. Each inpatient’s electronic patient record was reviewed to determine whether the blood tests recommended in the Trust’s physical health policy were taken within 24 hours of admission and whether an ECG was done within 24 hours of admission, as recommended in the Trust policy. We also determined whether any female patient of reproductive age had a urine pregnancy test done on admission and whether a urine drug screen had been done if there was a suspicion of, or known use of, any illicit substances.

**Results:** 89 of the 160 (56%) inpatients had a full blood count done on admission. 90 (56%) inpatients had urea and electrolytes done, 93 (58%) had liver function tests done, 90 (56%) had thyroid function tests done, 83 (52%) had an HbA1c level done, 89 (56%) had a random serum total cholesterol level and random lipid profile done. 86 (54%) of the 160 inpatients had an ECG done on admission. Of the 69 female inpatients, only 15 (22%) had a urine pregnancy test done. Only 31 (19%) of the 160 inpatients had a urine drug screen done.

**Conclusion:** This audit highlighted that current practice indicates need for improvement. There needs to be increased awareness of which blood tests are required following admission. This could be achieved during the junior doctor Trust induction. There is also a need to make nursing staff aware of the need for female inpatients of reproductive age to be offered a urine pregnancy test. There should be a daily review of any patients who decline any physical health investigations on admission, with attempts made to complete these as promptly as possible.

